# Granulation tissue-type hemangioma of ureter: a highly misdiagnosed disease (a rare case report and literature review)

**DOI:** 10.1186/s12894-022-01010-x

**Published:** 2022-04-19

**Authors:** Jiang Zhao, Xiaoqing Wang, Chi Zhang, Mingze He, Ge Bian, Ming Zhang, Mingchuan Liu, Xupeng Mu, Kebang Hu

**Affiliations:** 1 Department of Urology, The First Hospital of Jilin University, Changchun, 130031 China; 2 Department of Urology, First Moscow State Medical University, Moscow, Russia 119991; 3 Scientific Research Center, China-Japan Union Hospital, Jilin University, Changchun, 130033 China

**Keywords:** Ureteral granulation tissue hemangiomas, Ureteral neoplasms, Hemangioma

## Abstract

**Background:**

Ureteral granulation tissue hemangiomas are rare benign vascular lesions, and they may be clinically asymptomatic or present with massive or recurrent hematuria. Sometimes hemangiomas are difficult to distinguish from malignant ureteral tumors, and most ureteral hemangiomas are confirmed by postoperative pathological examination. This article aims to present a case of granulation tissue-type hemangioma of the ureter and briefly review the current literature on this condition.

**Case presentation:**

A 30-year-old male patient presented with complaints of painless macroscopic hematuria for 2 months. Computerized tomography of the urinary system showed that the upper 1/3 of the right ureter was occupied, and then the possibility of tumor lesions was considered. The urine cytology showed occasional nuclear abnormalities and many light-stained crystals in urine. Because of suspicious radiological and cytological findings, the patient underwent the right ureteroscopy and the laparoscopic right ureteral mass resection. The postoperative pathological report showed that it was a mesenchymal tumor. The morphological and immunohistochemical staining was consistent with that of hemangioma, tending to granulation tissue hemangioma. After surgery, the patient was in a good state and recovered well at the last follow-up.

**Conclusions:**

Ureteral granulation tissue hemangiomas are an easily misdiagnosed disease. Intermittent painless hematuria is an important characteristic of this disease. Therefore, we suggest that unnecessary radical surgery can be avoided when clinicians consider the possibility of benign ureteral tumors during the evaluation.

## Background

Ureteral granulation tissue hemangiomas are rare benign vasculopathy that is prone to misdiagnose and mistreat due to the atypical clinical manifestations of this disease and lack of relevant knowledge. Granulation tissue-type hemangiomas are common tumor-like vascular lesion of the skin and mucous membranes of the oral cavity that has traditionally been considered reactive proliferative lesion rather than tumor process [1]. Granulation tissue-type hemangiomas occur upon various stimuli, such as chronic mild stimulation, traumatic injury, and hormonal factors [2–5]. They range in size from a few millimeters to several centimeters and can grow rapidly with frequent bleeding [2]. However, granulation tissue-type hemangioma does very rarely occur in the urinary system. Sometimes hemangiomas are difficult to distinguish from malignant ureteral tumors. Meanwhile, most ureteral hemangiomas are confirmed by postoperative pathological examination [6]. Here, we present a rare case of granulation tissue-type hemangioma in the upper 1/3 ureteral segment with hydronephrosis and hematuria. This report intends to enhance clinicians' understanding of the disease and establish a clear diagnosis in the future.

## Case presentation

**Fig. 1:**
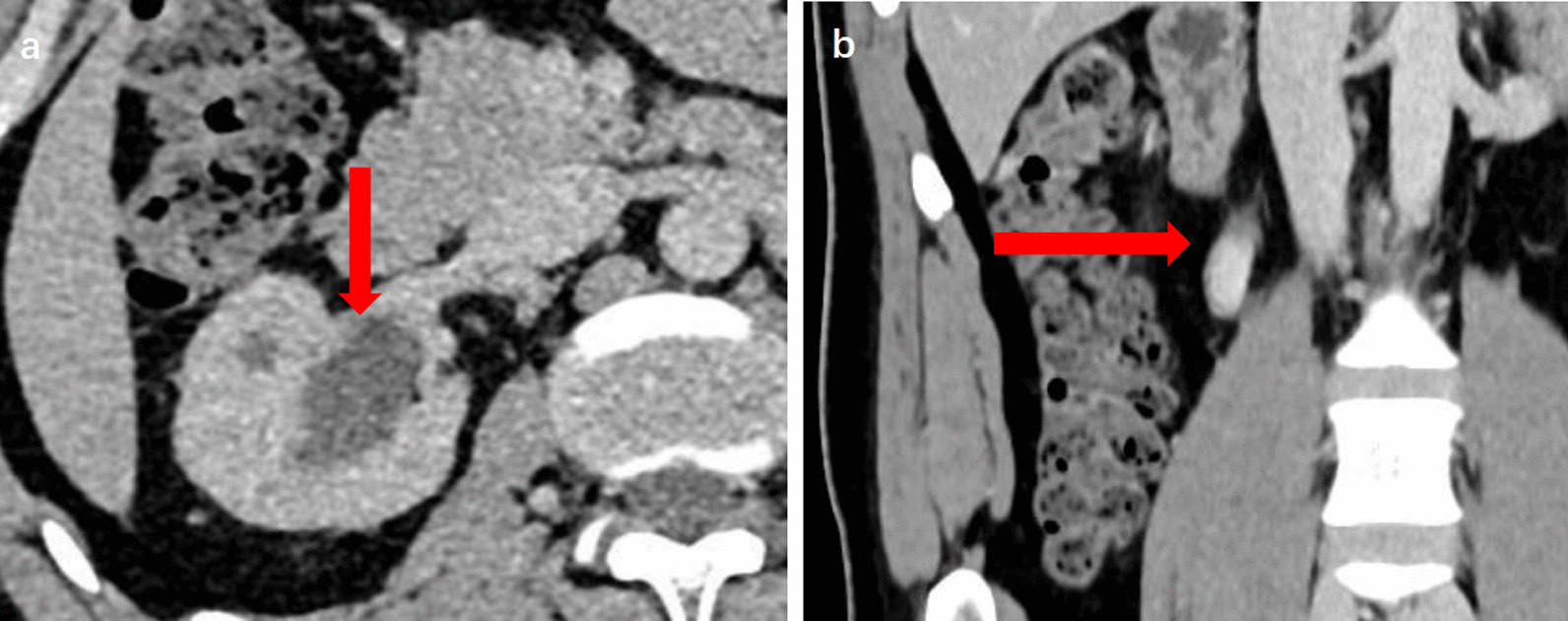
**a** Computerized tomography of the urinary system showed secondary right hydronephrosis, right renal parenchyma perfusion decreased (red arrow). **b** Computerized tomography of the abdomen showed the upper 1/3 of the right ureter was occupied and the possibility of tumor lesions was considered (red arrow)

**Fig. 2:**
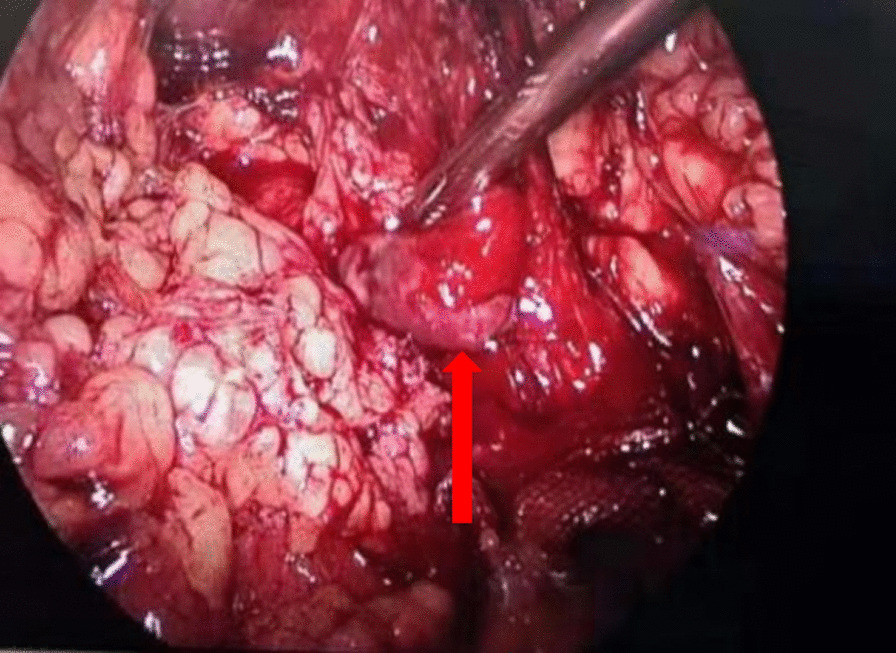
Ureteral tumors were seen under laparoscopy (red arrow)

**Fig. 3:**
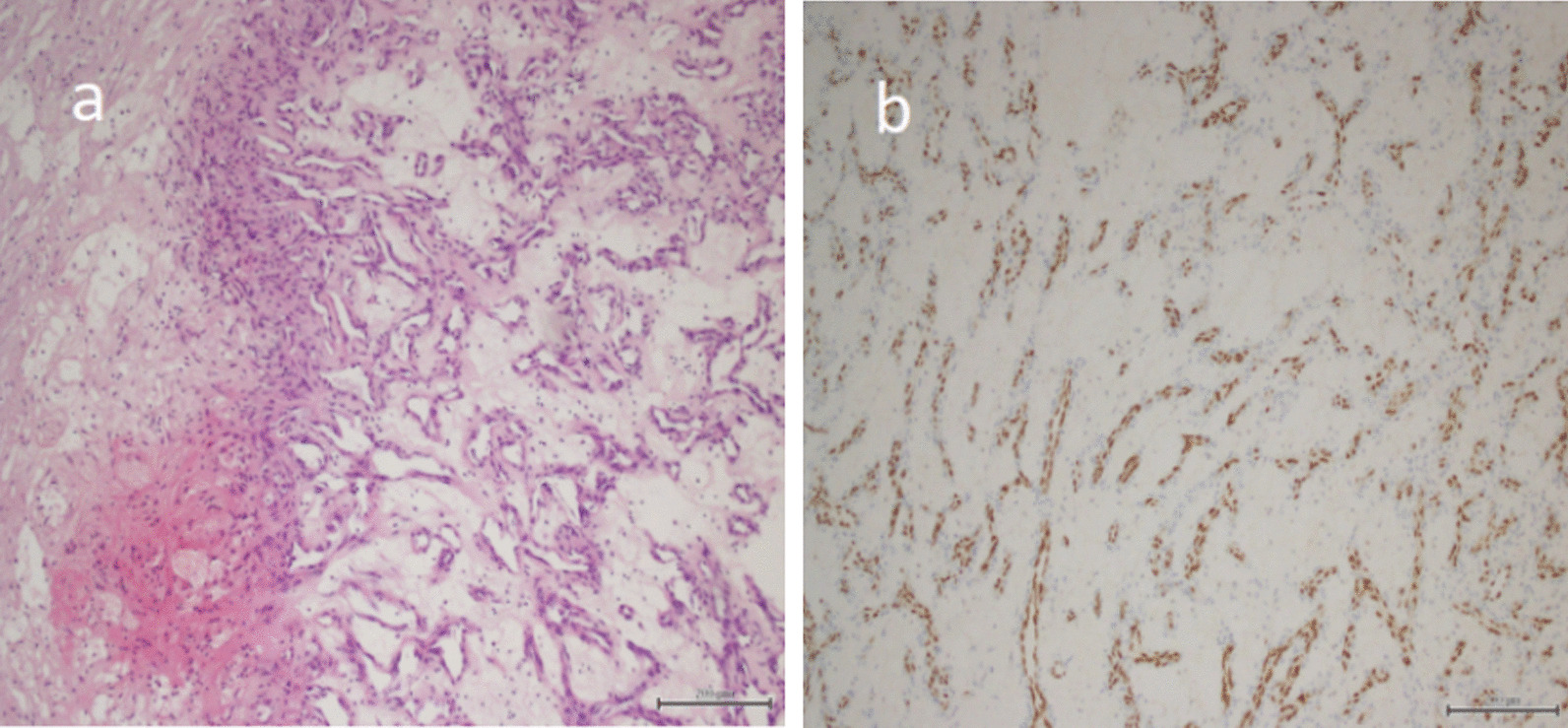
**a** At high-power magnification, capillaries are seen. The lumen is filled with numerous red blood cells. The interstitium is loose and edematous, surrounded by inflammatory exudate and necrotic tissue. **b** Immunohistochemistry suggested ERG (+)

**Fig. 4:**
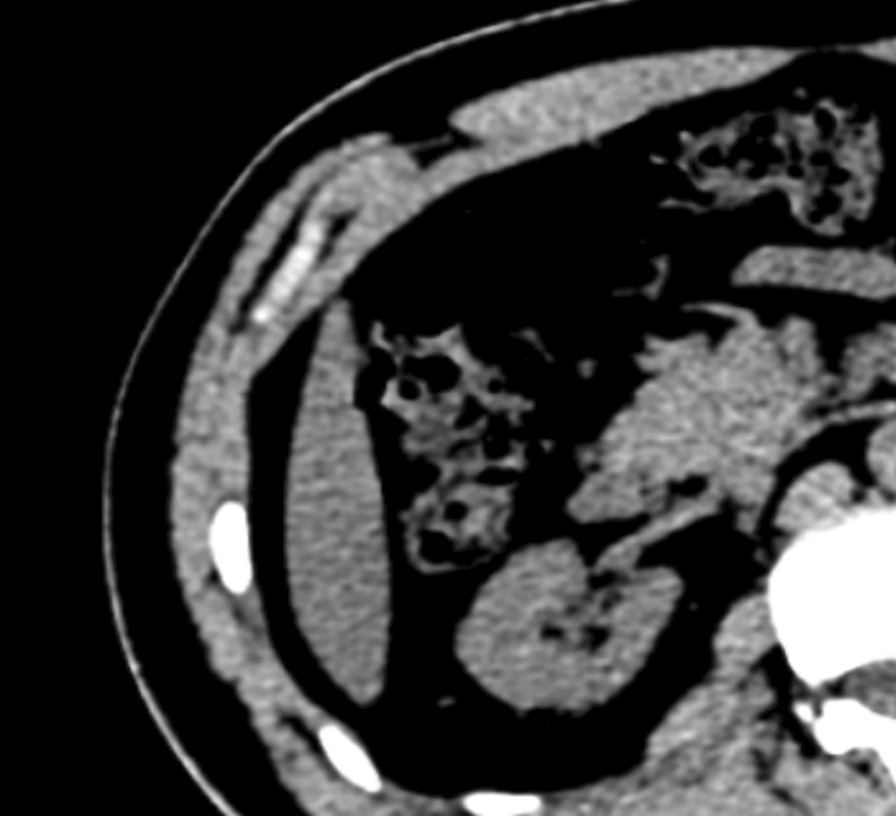
There was no dilated fluid in the right renal pelvis and ureteral lumen, and no recurrence of angioma

The patient was a 30-year-old male with painless macroscopic hematuria for 2 months. After examination, the patient had no hypertrophy of the prostate and normal sphincter tension. Meanwhile, the patient's external genitalia, bilateral spermatic cords, and testicles were normal. The urology color ultrasound showed hydronephrosis in the right kidney and dilation of the upper segment of the right ureteral canal. Intravenous urography showed no obvious abnormality in left renal function and morphology, decreased right renal function, and no obvious abnormality in the bladder. Computerized tomography of the urinary system showed that the upper 1/3 of the right ureter was occupied, and then the possibility of tumor lesions was considered. Meanwhile, the results showed that the peripheral lymph nodes were slightly enlarged, secondary right hydronephrosis, right renal parenchyma perfusion decreased, and right perirenal exudative changed (Fig. 1). The urine cytology showed occasional nuclear abnormalities and many light-stained crystals in urine. Because of suspicious radiological and cytological findings, the patient underwent the right ureteroscopy and the laparoscopic right ureteral mass resection (Fig. 2). A mass was visible on the ureteral wall during ureteroscopy, and the ureteral wall was smooth. Under laparoscope, a longitudinal incision was made along the ureter, and the mass was cut off along the tumor's base. After removal of ureteral masses, intraoperative frozen sections were performed. The first intraoperative frozen section report presented that fibrous tissue, necrotic tissue, and inflammatory exudates were found in the tissue, and no tumor components were found. The second intraoperative frozen section report presented that the mass was a mesenchymal tumor, which need to wait for paraffin and immunohistochemical examination to further clarify the diagnosis. We performed ureteral mass resection rather than radical ureterectomy in this case because the tumor was superficial, no malignant tumor was found in the frozen section, and no stenosis was found in the urinary tract after resection. Following the removal of the mass, the ureteral stent was placed in the ureter and intermittently sutured with an absorbable suture. The postoperative pathological report showed that it was a mesenchymal tumor. The morphological and immunohistochemical staining was consistent with that of hemangioma, tending to granulation tissue hemangioma. Meanwhile, some regional interstitial edema, surface necrosis, inflammatory exudates, bleeding, and fibrous tissue hyperplasia were found in the focal area (Fig. 3). The patient recovered well from surgery, and the ureteral stent was removed one month later. Half a year after surgery, the patient reviewed a CT of the urinary system. The results showed there was no dilatory water accumulation in the right renal pelvis and ureteral canal, and no hemangioma recurrence (Fig. 4). And the patient recovered well at the last follow-up.

## Discussion and conclusions

Granulation tissue-type hemangioma was first identified by Poncet and Dor in 1897 who described it as a vascularised mass and named it “Human Botryomycosis” [5]. And the more commonly used “pyogenic granuloma” was proposed by Hartzell in 1904. In 1980, Mills et al. proposed the term “lobular capillary hemangioma”, which provided a more meaningful name for all angioproliferative variants known as suppurative granuloma in history [7, 8]. The granulation tissue-type lesion has been considered to be reactive to various stimuli and hyperproliferative vascular responses, rather than a true hemangioma [1, 2]. The pathogenesis of granulation tissue-type hemangioma may be associated with minor trauma, medication, hormonal changes, and the production of vascular endothelial growth factors [2–5].

Reviewing the current literature, there appears to be only one report of ureteral granulation tissue-type hemangioma. In that report, the patients took microscopic haematuria and loin pain as the main symptoms. Given the minimal number of reports on ureteral granulation tissue-type hemangioma, we could draw evidence from similar manifestations of the hemangioma within the bladder [9]. There were some reports of bladder granulation tissue-type hemangioma, but the bladder hemangiomas were still rare, representing only 0.6% of the bladder masses [1, 9, 10]. These hemangiomas had traditionally been classified as cavernous or capillary, with some authors arguing for the inclusion of an arteriovenous type. Of these, most hemangiomas were cavernous types, with the capillary subtype being far much rarer [9, 11]. In many cases of intravesical hemangiomas, the presence of intermittent gross hematuria often provides a guide to the final diagnosis [10]. In our case, hematuria is also the main symptom of the patient.

As part of the diagnostic evaluation for haematuria, a polypoid or sessile lesion is found incidentally extending from the urothelium, either on contrast-CT scanning as a filling defect or endoscopically [1, 9, 10, 12]. Even though radiological and histopathological findings can aid in the diagnosis and treatment of sarcoid-type hemangiomas, we believe that we cannot adequately characterize the nature of the mass from imaging and that excisional biopsies are needed to exclude the possibility of more serious disease because certain histological features help to distinguish sarcoid-type hemangiomas from other lesions [2]. Although spontaneous regression of granulomatous hemangiomas at other sites has been reported, surgical excision is often the reported option due to their propensity for bleeding and ulceration [8, 13]. Therefore, we also took a surgical resection method for this ureteral granulation tissue-type tumor. In addition, surgical resection is the first choice for histopathological diagnosis, which is helpful to exclude malignant lesions [7, 13–15].

In our case, the laparoscopic right ureteral mass resection was performed and the patient remained well for more than six months following surgery, with no evidence of recurrence. There are no reliable follow-up data for haemangiomas within the urinary tract. Once diagnosed, CT and ureteroscopy could be performed during follow-up. The patient recovered well and had a good prognosis during the last follow-up. This is a rare case of granulation tissue-type hemangioma, and the excision biopsy is necessary for diagnosis. However, because of the relatively high incidence of ureteral malignancies, the choice of treatment is difficult and challenging.

In Conclusion, granulation tissue-type hemangiomas are rare benign vascular lesions, which mainly affect the skin and oral mucosa. Usually, it is confirmed by pathological examination. However, urinary cytology, ureteroscopy, and intraoperative frozen edge examination can guide surgeons to perform more conservative surgery, but it is necessary to keep in mind the benign nature of the mass. We performed ureteral mass resection and the patient recovered well at follow-up. Therefore, we recommend that clinicians avoid unnecessary radical surgery when evaluating the possibility of benign ureteral tumors.

## Data Availability

The data and material used or analysed during the current study are available from the corresponding author on reasonable request.

## Abbreviation

CT: Computed tomography
